# Melatonin-induced restoration of the intestinal mucosal barrier in inflammatory bowel disease via activation of the SIRT1-LKB1-pAMPK axis

**DOI:** 10.3389/fimmu.2026.1811583

**Published:** 2026-05-05

**Authors:** Qingyi Mao, Beibei Lin, Wenhao Xia, Yu Zhang, Yue Lei, Qian Cao, Mengque Xu

**Affiliations:** 1 Department of Gastroenterology, Sir Run Run Shaw Hospital, College of Medicine Zhejiang University, Hangzhou, China; 2 Inflammatory Bowel Disease Center, Sir Run Run Shaw Hospital, College of Medicine Zhejiang University, Hangzhou, China

**Keywords:** gut microbiota, inflammatory bowel disease, intestinal mucosal barrier, melatonin, SIRT1-LKB1-pAMPK axis

## Abstract

**Background:**

Numerous studies indicated inflammatory bowel disease (IBD) patients suffered from sleep disturbances. Although melatonin (MT) exerts positive effects on maintaining circadian rhythms and anti-inflammation, its impact on the gut microbiome and its function in mediating gut health remain largely unexplored. To evaluate the efficacy and investigate the mechanisms of MT in repairing the intestinal mucosal barrier in IBD.

**Methods:**

Fecal MT and its metabolites in IBD patients were detected. A DSS-induced colitis mice model and a LPS-stimulated NCM460 cell inflammation model were used to explore the mechanism of melatonin in IBD.

**Results:**

IBD patients had lower levels of serum MT and fecal 2-oxomelatonin. Furthermore, MT enhances intestinal antimicrobial peptides and effectively alleviates colitis. Mechanistically, MT restored the abundance of the probiotic *Akkermansia* and decreased the conditional pathogen *Desulfovibrio*. MT upregulates the SIRT1 (Sirtuin 1) and pAMPK (phosphorylated AMP-Activated Protein Kinase) in mouse colonic tissues. Whereas Ex-527 (the SIRT1 inhibitor) and Compound C (the pAMPK inhibitor) abolished the protective effects of MT in DSS mice. In LPS-stimulated cells, the inhibitor blocked the regulation of MT on proinflammatory factors, antimicrobial peptides and tight junctions. Mechanistically, MT was associated with activation of the SIRT1-LKB1-pAMPK pathway, suggesting its potential involvement in regulating the above changes.

**Conclusion:**

Our findings suggest that MT may ameliorate colitis by regulating gut microbiota, modulating antimicrobial peptide secretion, and reinforcing intestinal epithelial barrier integrity potentially via activation of the SIRT1-LKB1-pAMPK axis.

## Graphical Abstract

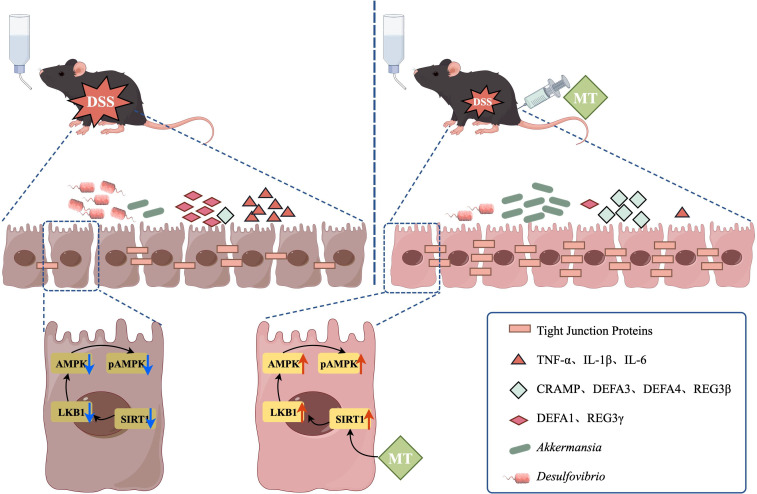

## 1 Introduction

Inflammatory bowel disease (IBD) is a chronic inflammatory condition of the gastrointestinal tract, encompassing Crohn’s disease (CD) and ulcerative colitis (UC) (1, 2). Sleep disturbances are prevalent among IBD patients (3–5), affecting even pediatric and adolescent patients (6). The incidence of sleep disturbances among IBD patients in China is 60.2% (7). Epidemiological research indicates a synchronicity between the disease activity of IBD and risk increase of IBD relapse (3–5). Studies have documented that sleep disturbances may be an extraintestinal manifestation of IBD, with IBD patients experiencing less rapid eye movement (REM) sleep and more microarousals compared to healthy controls (8, 9). Evidence has emerged that there is a complex bidirectional relationship between sleep disturbances and IBD, where sleep issues may be both a consequence and a potential cause of IBD (10, 11). However, the links between circadian rhythm disturbance and the disorder in intestinal homeostasis remain incompletely understood.

Melatonin (MT), a hormone synthesized in the pineal gland, plays a crucial role in regulating the sleep-wake cycle (12, 13). MT has been recognized for its potential improvement in sleep quality (14), and is widely used in treating insomnia (15, 16). Apart from regulating sleep rhythms, MT also has anti-inflammatory and antioxidant effects (16). Additionally, it is worth noting that MT levels in the human gut are 400 times higher than in the pineal gland (17, 18). Clinical evidence suggests that UC patients exhibit reduced plasma MT levels (19). Nevertheless, the impacts and underlying mechanisms of additional MT in maintaining intestinal homeostasis remain uninvestigated.

SIRT1, a member of the sirtuin family, is a nicotinamide adenine dinucleotide (NAD^+^)-dependent deacetylase (20). It regulates cellular metabolism, oxidative stress, immune inflammation, cell cycle, apoptosis, and senescence by removing acetyl groups from proteins such as Nuclear Factor kappa B (NF-κB) and signal transducer and activator of transcription 3 (STAT3) (21). Phosphorylated AMP-activated protein kinase (pAMPK) is a cellular energy sensor that promotes energy production and maintains energy homeostasis by regulating glucose and lipid metabolism. Additionally, it participates in diverse biological processes, including autophagy, inflammatory responses, and oxidative stress. pAMPK can be primarily activated by three upstream kinases: liver kinase B1 (LKB1), calcium/calmodulin-dependent protein kinase kinase β, and transforming growth factor-β-activated kinase 1 (21). SIRT1 and pAMPK exhibit intricate interactions. SIRT1 can activate LKB1, one of the primary upstream kinases of pAMPK, through deacetylation, while pAMPK may activate SIRT1 by upregulating NAD^+^ levels (22). Whether MT ameliorates colitis via activation of the SIRT1/pAMPK pathway remains unknown.

In this study, we observed that UC patients have less plasma MT levels and fecal MT metabolite. Subsequent MT supplementation restores the intestinal dysbiosis, enhanced the function of intestinal barrier, resulting in alleviation of colitis in mice. Mechanistically, MT upregulated the expression of intestinal LKB1-pAMPK signaling via SIRT-1, thereby enhancing intestinal barrier function and ameliorating the intestinal inflammation.

## 2 Methods

### 2.1 Clinical samples

For both serum and fecal samples, patients were selected from the IBD Center of Sir Run Run Shaw Hospital. For serum samples, a total of 12 patients with IBD who had not been previously treated with corticosteroids, immunosuppressants, or biologics (with the exception of mesalazine) were included, including 6 CD and 6 UC. Additionally, 6 healthy individuals without gastrointestinal diseases, matched for age and sex, were also included as healthy controls (HCs). For fecal samples, 32 IBD patients (21 CD and 11 UC) and 26 HCs were enrolled using the same inclusion criteria. Importantly, Patients with comorbidities or a history of melatonin or other sleep medication use within three months prior to enrollment were excluded. The clinical characteristics of IBD patients and NCs are presented in (**Supplementary Tables 1**, **2**). Fecal and serum samples from all individuals were collected under a protocol approved by the Institutional Ethics Committee of Sir Run Run Shaw Hospital (approval No. 2025-4052-01).

### 2.2 Enzyme-linked immunosorbent assay

A commercial human melatonin ELISA kit (purchased from Thermo Fisher Scientific, USA, catalog number ml856212-J) was used to detect the levels of melatonin in the serum of patients and healthy control subjects, following the manufacturer’s protocol.

### 2.3 Cell culture and induction of inflammation

NCM460 (ORC0841) was obtained from Oricells Biotechnology (Shanghai, China). NCM460 cells were maintained in RPMI1640. The culture medium was supplemented with 10% fetal bovine serum (FBS) and penicillin-streptomycin at a concentration of 1×. All cells were cultivated in a humidified environment at 37 °C with 5% CO2. LPS stimulation was employed as it directly activates Toll-like Receptor 4 (TLR4) signaling in intestinal epithelial cells and has been widely used to model epithelial inflammation in IBD-related studies (23). Cells were pretreated with 1 mM MT (HY-B0075, Med Chem Express, USA) in culture medium for 24 hours, followed by stimulation with 1 μg/ml LPS for 24 hours to induce inflammation. For inhibition experiments, 0.5 μM Ex-527 (HY-15452, Med Chem Express, USA; a SIRT1 inhibitor) or Compound C (HY-13418A, Med Chem Express, USA; an pAMPK inhibitor) was co-administered with MT in the culture medium.

### 2.4 Animal experiment

All male C57BL/6 mice (6 weeks old) were housed in a specific pathogen free (SPF) level facility at the Animal Experimental Center of Sir Run Run Shaw Hospital, Zhejiang University. The housing conditions were maintained at a temperature of 20 °C ± 2 °C, with relative humidity of 50%-60%, and a 12/12-hour light/dark cycle. Mice had free access to food and water throughout the experiment. Mice were allocated into groups according to the experimental design: Normal Control (NC) group, Colitis (DSS) group, Melatonin Treatment (DSS+MT) group, and respective inhibitor groups (DSS+MT+Ex, DSS+MT+Cc). MT was administered at a dosage of 10 mg/kg/day via intraperitoneal injection. Ex-527 was administered at a dosage of 2 mg/kg/day via intraperitoneal injection. Compound C was administered at a dosage of 5 mg/kg/day via intraperitoneal injection. An acute colitis model was induced using 2% DSS in drinking water. MT, Ex-527, and Compound C were administered 5 days prior to DSS treatment and continued throughout the experimental period. Disease severity was assessed daily. When the mice exhibited approximately 15% body weight loss, they were deeply anesthetized with 4-5% isoflurane in 100% O_2_ (1 L/min) by inhalation, and then sacrificed by cervical dislocation immediately. The colorectum from the cecum to the anus was collected. The distal 5 mm of the colon was used for H&E staining, and the whole remaining colon tissue was homogenized for RNA or protein extraction.

### 2.5 Histopathology experiment

Colonic tissues proximal to the anus of the mice were harvested and fixed in 4% paraformaldehyde. The tissues were then embedded and sectioned into 5μm slices. Staining was performed using Hematoxylin and Eosin (H&E) staining. Immunofluorescence was initiated by deparaffinizing the colonic sections with alcohol and xylene, followed by incubation in a trypsin repair solution for 1 hour. The sections were then blocked with 5% bovine serum albumin (BSA) for 30 minutes. After removal of the blocking solution, the sections were incubated with the primary antibody overnight at 4 °C (ZO-1 antibody diluted at 1:2000). After washing, the sections were incubated with the secondary antibody for 60 minutes at room temperature, and then stained with DAPI and an anti-fade mounting medium.

### 2.6 Quantitative real-time PCR

Total RNA was extracted from mice colon and NCM460 cells, followed by reverse transcription. Primers were designed based on sequences specific to mice and humans and synthesized by Tsingke, China. A summary of all primer names and sequences is provided in the **Supplementary Table 3**. qPCR was performed in a 10 μl reaction system containing specific primers and SYBR premix (Vazyme Biotech, Nanjing, China). Amplification was carried out using an ABI StepOne plusTM RT-PCR system (Carlsbad, CA). The reaction protocol was set as follows: 95 °C for 5 min (initial denaturation), 95 °C for 10 s, 60 °C for 30 s (PCR cycling), for a total of 40 cycles. mRNA levels were normalized to Gapdh relative RNA expression was analyzed using the 2−ΔΔCt method.

### 2.7 Western blotting

Colonic segments were rapidly homogenized in RIPA lysis buffer, centrifuged at 12,000 g for 10 minutes at 4 °C, and the supernatant was collected and stored at -80 °C for Western blotting analysis. Protein concentrations were determined using a Bicinchoninic Acid (BCA) assay kit. Twenty micrograms of protein samples were subjected to electrophoresis on a 10% sodium dodecyl sulfate-polyacrylamide gel. Samples were electrotransferred onto a polyvinylidene fluoride (PVDF) membrane, blocked with 5% skim milk in 1× Tris-buffered saline with Tween (TBST) for 2 hours at room temperature, and incubated with Polyclonal Antibodies (GAPDH, 1:2000; Tublin, 1:2000; Actin 1:2000; Occludin, 1:5000; SIRT1, 1:5000; pAMPK, 1:5000; LKB1, 1:2000) overnight at 4 °C. After washing with TBST, membranes were incubated with horseradish peroxidase-conjugated goat anti-rabbit/mouse IgG (1:2000) at 37 °C for 1 hour. Data analysis was performed using Image Lab.

### 2.8 16S rDNA amplicon sequencing

16S rDNA amplicon sequencing was performed by Shanghai Realbio Technology Co., Ltd., China. The V3-V4 hypervariable regions of 16S rDNA genes were amplified and sequenced. Paired-end reads were assembled using Pandaseq v2.9, with low-quality bases trimmed from both ends. Sequences were filtered by length (250–500 nt), excluding those with average quality scores <20 or containing >3 ambiguous bases (N). Singletons were removed, and operational taxonomic units (OTUs) were clustered at 97% similarity threshold using USEARCH v7.0.1090 followed by chimera filtering. Taxonomic classification was performed by comparing OTUs against the RDP Classifier (https://sourceforge.net/projects/rdp-classifier/). Alpha diversity indices were calculated using QIIME v1.9.1, with rarefaction curves generated to verify data sufficiency. Principal coordinates analysis (PCoA) was conducted using the dudi.pco function in the ad4 package of R. Permutational multivariate analysis of variance (PERMANOVA/Adonis) was performed using the adonis function in the vegan package. OTU abundances were aggregated at phylum and genus levels for stacked bar plot visualization. Heatmaps were generated to display relative abundances at genus level.

### 2.9 Untargeted metabolomics sequencing of human fecal samples

Untargeted metabolomics sequencing of human fecal samples was performed by Hangzhou Cosmos Wisdom Biotechnology Co., Ltd., China. It was performed on human fecal specimens using high-resolution mass spectrometry (HRMS) coupled with liquid chromatography (LC) separation. Metabolites were detected in both positive and negative ionization modes with full scan acquisition. Raw data were processed using MS-DIAL for feature extraction, peak alignment, and compound identification against reference databases (HMDB, KEGG). Multivariate statistical analysis was conducted using CV, PCA, PCC and etc.

### 2.10 Statistical analysis

Statistical analysis and data visualization were performed using GraphPad Prism 10. Quantitative data are presented as mean ± standard deviation (SD). Differences between two groups were analyzed using Student’s t-test, while comparisons among multiple groups were assessed by one-way ANOVA followed by Tukey’s multiple comparisons test. A *p* value of less than 0.05 was considered statistically significant.

## 3 Results

### 3.1 MT levels were reduced in IBD patients compared with healthy controls

**Figure 1:**
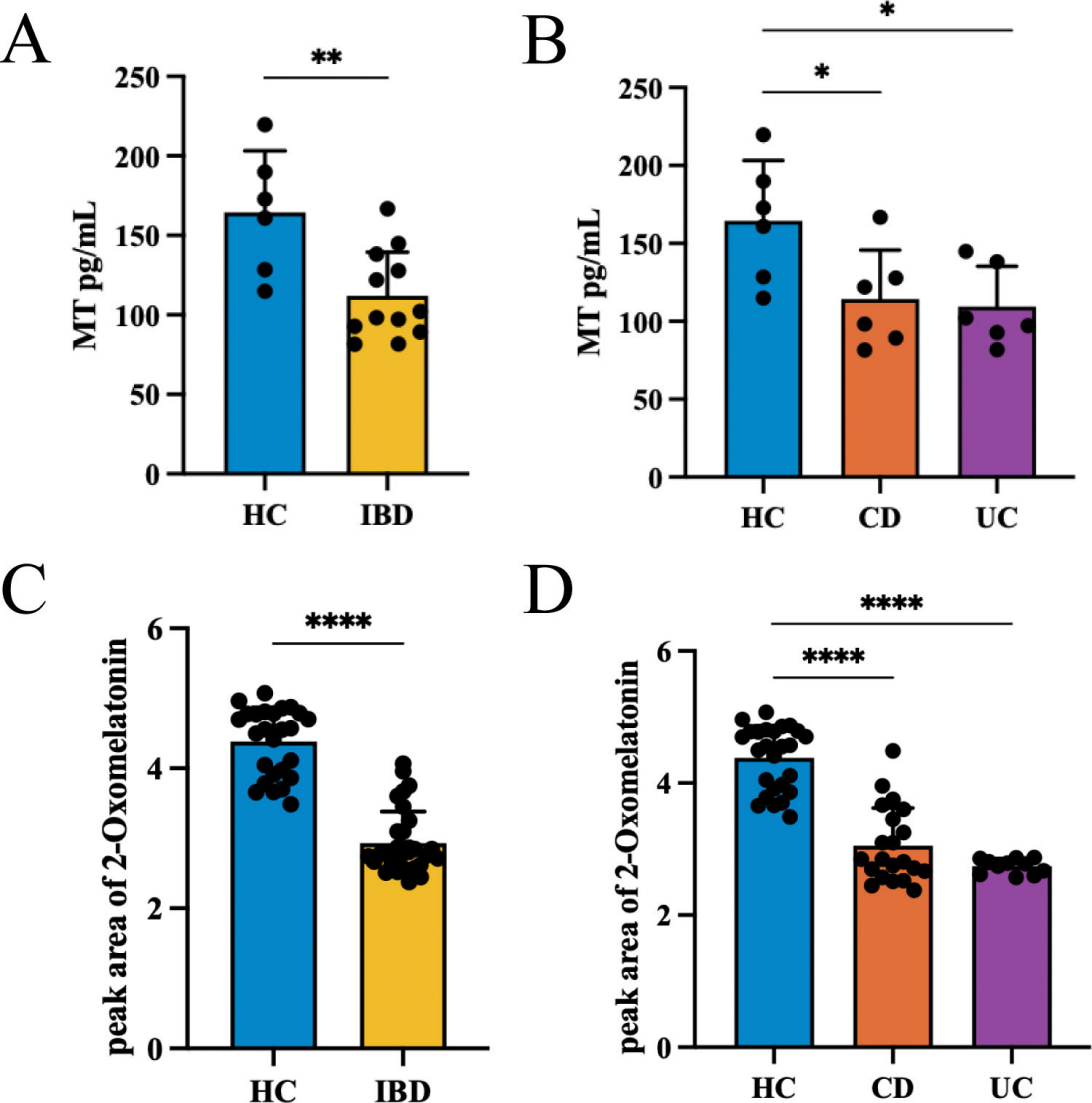
Decreased melatonin and its derivatives in IBD patients. **(A, B)** Serum MT: n=6 (HC), n=12 (IBD), n=6 (CD), n=6 (UC). **(C, D)** Fecal 2-oxomelatonin: n=26 (HC), n=32 (IBD), n=21 (CD), n=11 (UC). (*p < 0.05, **p < 0.01, ****p < 0.0001).

To investigate the MT levels in IBD patients, we collected serum samples from 12 IBD patients (including 6 CD and 6 UC) and 6 healthy controls (HCs), and measured MT levels by ELISA. Compared with the HC group, the serum MT levels in the IBD group were significantly decreased. Similar results were observed when separately comparing the CD group and the UC group (HC 164.5 ± 38.84 pg/ml vs. CD 114.3 ± 31.46 pg/ml vs. UC 109.5 ± 25.81 pg/ml) (**Figures 1A,B**). MT undergoes diverse metabolic pathways *in vivo*, being oxidized by hepatocytes into 2-oxomelatonin, which is then excreted into the intestine via bile and ultimately eliminated in feces (24). To further elucidate the intestinal metabolic characteristics of MT in IBD patients, we collected fecal samples from 32 IBD patients (including 21 CD and 11 UC) and 26 HCs for fecal untargeted metabolomics sequencing. Compared with the HC group, the IBD group exhibited a significant reduction in 2-oxomelatonin levels. Consistent results were observed in separate comparisons between the CD group and the UC group (HC 4.380 ± 0.4865 vs. CD 3.054 ± 0.5701 vs. UC 2.744 ± 0.1116) (**Figures 1C,D**).

### 3.2 MT ameliorates intestinal inflammation and mucosal barrier dysfunction

**Figure 2:**
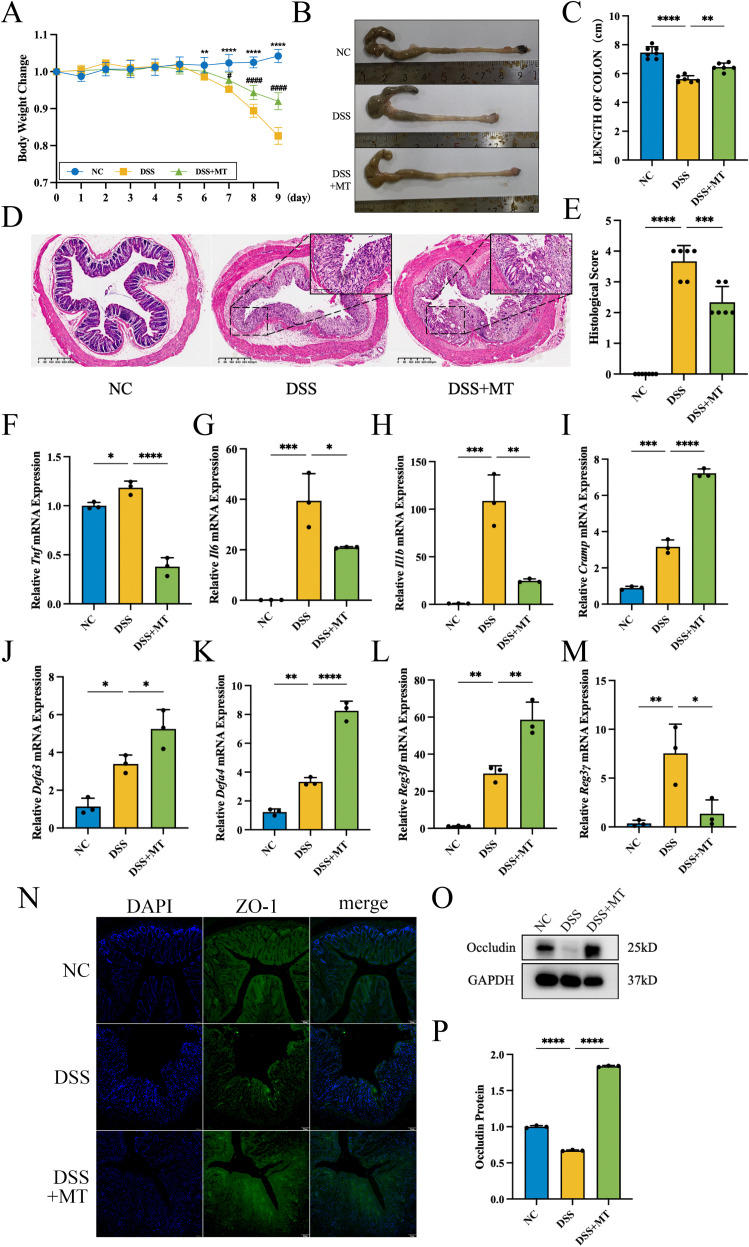
MT attenuated the DSS-induced inflammation in mouse colon. For body weight, colon length, and H&E histopathological scoring: n=7 (NC), n=6 (DSS), n=6 (DSS+MT). For qPCR and Western blotting analyses: n=3 per group. **(A)** Body weight changes of mice. (* indicates NC group compared to DSS group, # indicates DSS+MT group compared to DSS group.) **(B, C)** The length of mice colon. **(D, E)** H&E staining of colon tissues and histopathological scoring. Scoring criteria (0-4): 0, No inflammation; 1, Mild inflammation (1–2 mononuclear cell infiltrates); 2, Moderate inflammation (multiple infiltrates); 3, Severe inflammation (marked wall thickening, increased vascularity); 4, Transmural inflammation (granulocyte infiltration, goblet cell loss). **(F–H)** The relative mRNA expression levels of proinflammatory factors. **(I–M)** The relative mRNA expression levels of antimicrobial peptides. **(N)** The image of ZO-1 immunofluorescence. The green color showed ZO-1, and the blue color showed cell nuclear. **(O, P)** The image of WB of Occludin protein. (*p < 0.05, **p < 0.01, ***p < 0.001, ****p < 0.0001; #*p* < 0.05, ####*p* < 0.0001).

To further investigate the specific mechanism of MT as a therapeutic agent to improve IBD, we established a DSS-induced colitis mice model and administered MT intervention. As shown in **Figures 2A–E**, MT intervention significantly reversed the weight loss trend in DSS-induced colitis mice. The colon length in the DSS+MT group also showed marked recovery compared to the DSS group. Compared with the NC group, the DSS group exhibited significant pathological alterations in colon HE staining, including crypt structure destruction and inflammatory cell infiltration. Although the DSS+MT group still displayed some tissue damage, it demonstrated preserved villus integrity and partial crypt morphology compared to the DSS group. MT intervention significantly reduced the histopathological score.

We further evaluated the transcriptional levels of pro-inflammatory cytokines and antimicrobial peptides in colon tissues. As shown in **Figures 2F–H**, the DSS group demonstrated significantly elevated expression levels of pro-inflammatory cytokines (*Tnf*, *Il1b*, and *Il6*), which were markedly reduced following MT treatment. As shown in **Figures 2I–M**, compared to the NC group, the DSS group showed significantly elevated expression levels of Cathelicidin-related antimicrobial peptide (*Cramp*), *Defa3, Defa4, Reg3β*, and *Reg3γ*. Given that different antimicrobial peptides possess distinct physiological functions, the DSS+MT group exhibited further upregulation of *Cramp*, *Defa3*, *Defa4* and *Reg3β*, while *Reg3γ* expression was significantly downregulated.

To investigate whether intestinal mucosal barrier function was associated with MT treatment, immunofluorescence staining and WB analysis were performed to assess tight junction protein expression as structural markers of barrier integrity. Compared with the NC group, the DSS group exhibited reduced expression of ZO-1 protein (green fluorescence) in the colonic epithelium, while MT intervention was associated with partially restored ZO-1 expression (**Figure 2N**). As demonstrated in **Figures 2O,P**, Occludin relative expression was significantly reduced in the DSS group but was higher after MT treatment. These observations indicate an association between MT treatment and improved tight junction protein expression, although causality cannot be inferred from these data alone.

**Figure 3:**
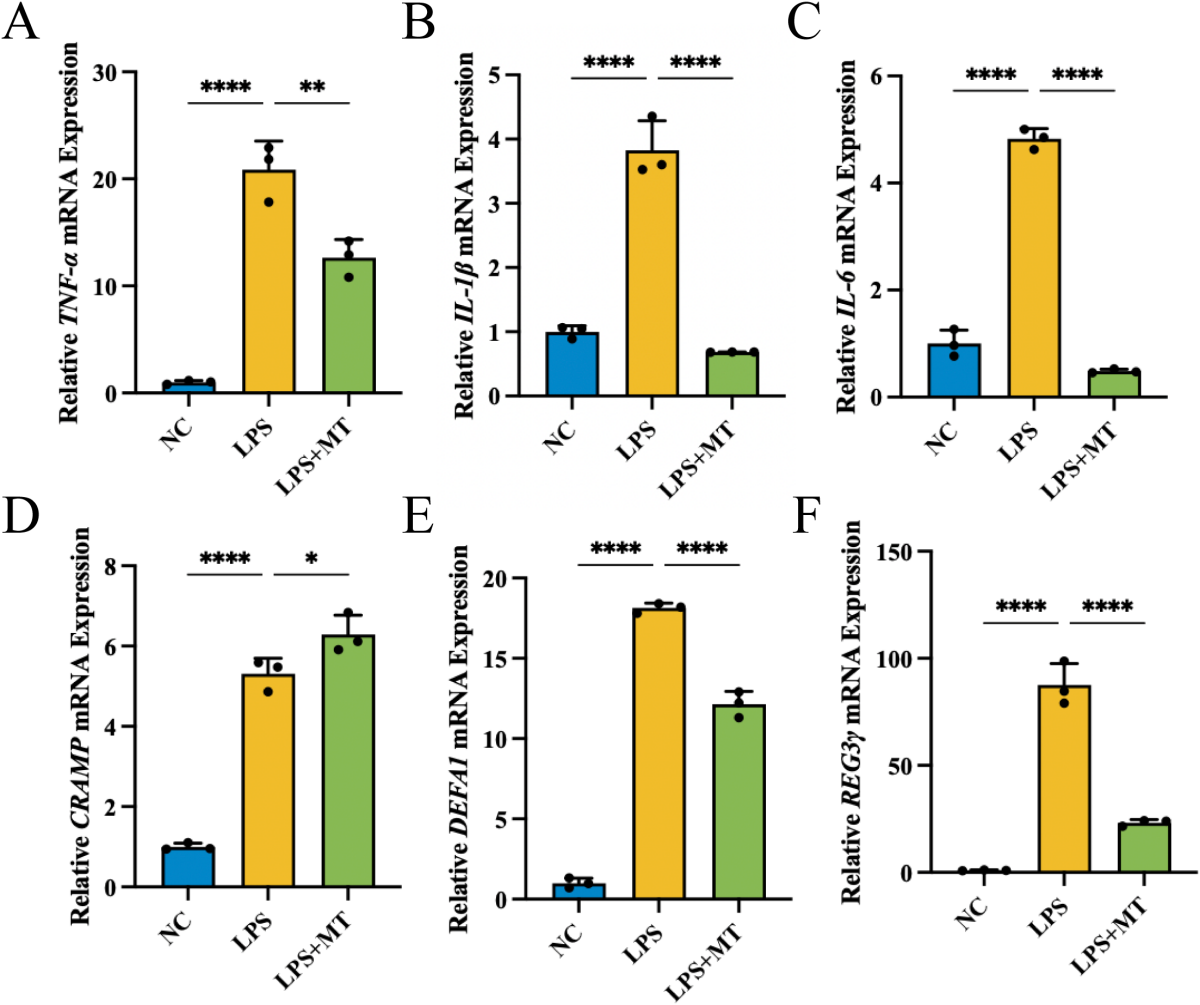
MT regulated the cytokines and antimicrobial peptides *in vitro*, n=3 (all groups). **(A–C)** The relative mRNA expression levels of proinflammatory factors. **(D–F)** The relative mRNA expression levels of antimicrobial peptides. (*p < 0.05, **p < 0.01, ****p < 0.0001).

In order to exclude the complex environmental interference *in vivo*, MT was used to intervene in the LPS inflammation model of NCM460 cells. As shown in **Figures 3A–C**, the levels of transcript for pro-inflammatory factors (*TNF-ɑ*, *IL-1β* and *IL-6*) were enhanced following LPS stimulation, which was significantly inhibited by MT pretreatment. As shown in **Figures 3D–F**, the levels of *CRAMP*, *DEFA1* and *REG3γ* were significantly elevated in the LPS-stimulated group compared with NC group. Following MT intervention, *CRAMP* expression was further upregulated, whereas the elevated levels of *DEFA1* and *REG3γ* were significantly reduced in the LPS+MT group.

### 3.3 MT reverses gut microbiota dysbiosis in DSS-colitis mice

**Figure 4:**
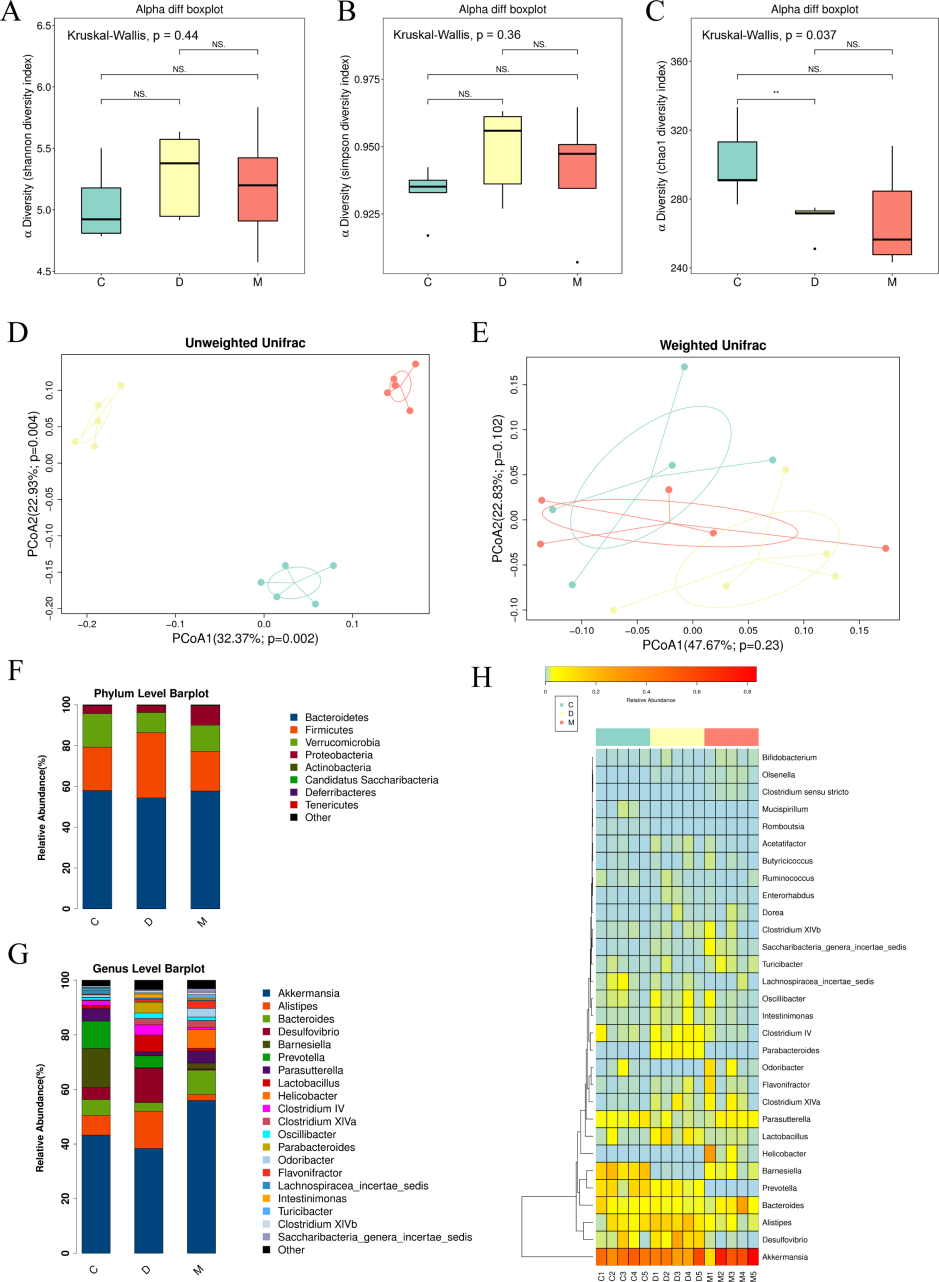
MT altered the gut microbiota community in DSS-induced colitis, n=5 (all groups). **(A–C)** α-diversity indices of mice gut microbiota. **(D, E)** β-diversity indices of mice gut microbiota. **(F)** The stacked bar plot of relative abundance at the phylum level. **(G)** The stacked bar plot of relative abundance at the genus level. **(H)** The heatmap of genus-level relative abundances. (C/green - NC group, D/yellow - DSS group, M/red - DSS+MT group) (ns *p*>0.05, **p < 0.01).

The feces of mice were collected when the mice had lost approximately 10% of their body weight. 16S rDNA amplicon sequencing was then performed. As shown in **Figures 4A–C**, both the Shannon and Simpson diversity index exhibited elevated levels in the DSS group. Following MT treatment, a decline in these indices was observed. However, this tendency did not attain statistical significance. The Chao1 diversity index demonstrated a substantial decrease in the DSS group relative to the NC group, and this difference was found to be statistically significant. Subsequent analysis was conducted to assess the β-diversity of the intestinal flora. The results of the unweighted PCoA analysis demonstrated a tendency for significant separation of the three groups on the PCoA1 and PCoA2 axes, indicating potential differences in species composition among the three groups (**Figure 4D**). Weighted PCoA analyses indicated the presence of some differences among the three groups. However, these differences did not attain statistical significance. In comparison with the DSS group, there was a greater degree of overlap between the NC and DSS+MT groups, suggesting that the NC and DSS+MT groups differed to a comparatively lesser extent in terms of community abundance (**Figure 4E**).

In the taxonomic analysis of gut microbiota, the community structure analysis based on the level of phylum showed that the dominant phylum were *Bacteroidetes, Firmicutes, Verrucomicrobia* and *Proteobacteria*. The relative abundance of *Firmicutes* was increased in the DSS group, while the abundance was similar between the NC and DSS+MT groups. Conversely, the DSS group exhibited a decline in the abundance of *Verrucomicrobia*, indicative of potential suppression, which was followed by a recovery post-MT intervention. However, none of the differences in the above were statistically significant (**Figure 4F**).

The relative abundance at the genus level was further assessed. As shown in **Figures 4G, H** and **Supplementary Figures 1A, B**, *Akkermansia* exhibited a decreasing trend in DSS group and an increasing trend in DSS+MT group, but without statistical significance(NC 43.30 ± 6.203 vs. DSS 38.38 ± 12.98 vs. DSS+MT 55.91 ± 27.65). Furthermore, the DSS group exhibited a higher abundance of *Desulfovibrio* compared to the NC group, but without significance (NC 4.398 ± 6.380 vs. DSS 12.58 ± 7.398, *p* > 0.05). Following MT intervention, *Desulfovibrio* levels were markedly reduced, even below the baseline levels observed in the NC group (DSS+MT 0.3985 ± 0.4634, *p* = 0.0132 vs. DSS).

To explore the functional relevance of the observed microbial shifts, correlation analyses were performed between the relative abundances of key genera and markers of intestinal inflammation and barrier function. As shown in **Supplementary Figures 2A, B**, the abundance of *Akkermansia* was negatively correlated with colonic *Tnf* mRNA levels, while *Desulfovibrio* abundance showed a positive correlation with *Tnf* and *Il6* mRNA levels. Additionally, *Akkermansia* abundance was positively correlated with Occludin expression, whereas *Desulfovibrio* showed a negative correlation (**Supplementary Figures 2C–E**). These correlations suggest a potential link between microbiota composition and intestinal inflammation and barrier integrity.

### 3.4 MT upregulates SIRT1 and pAMPK expression in DSS-colitis mice

**Figure 5:**
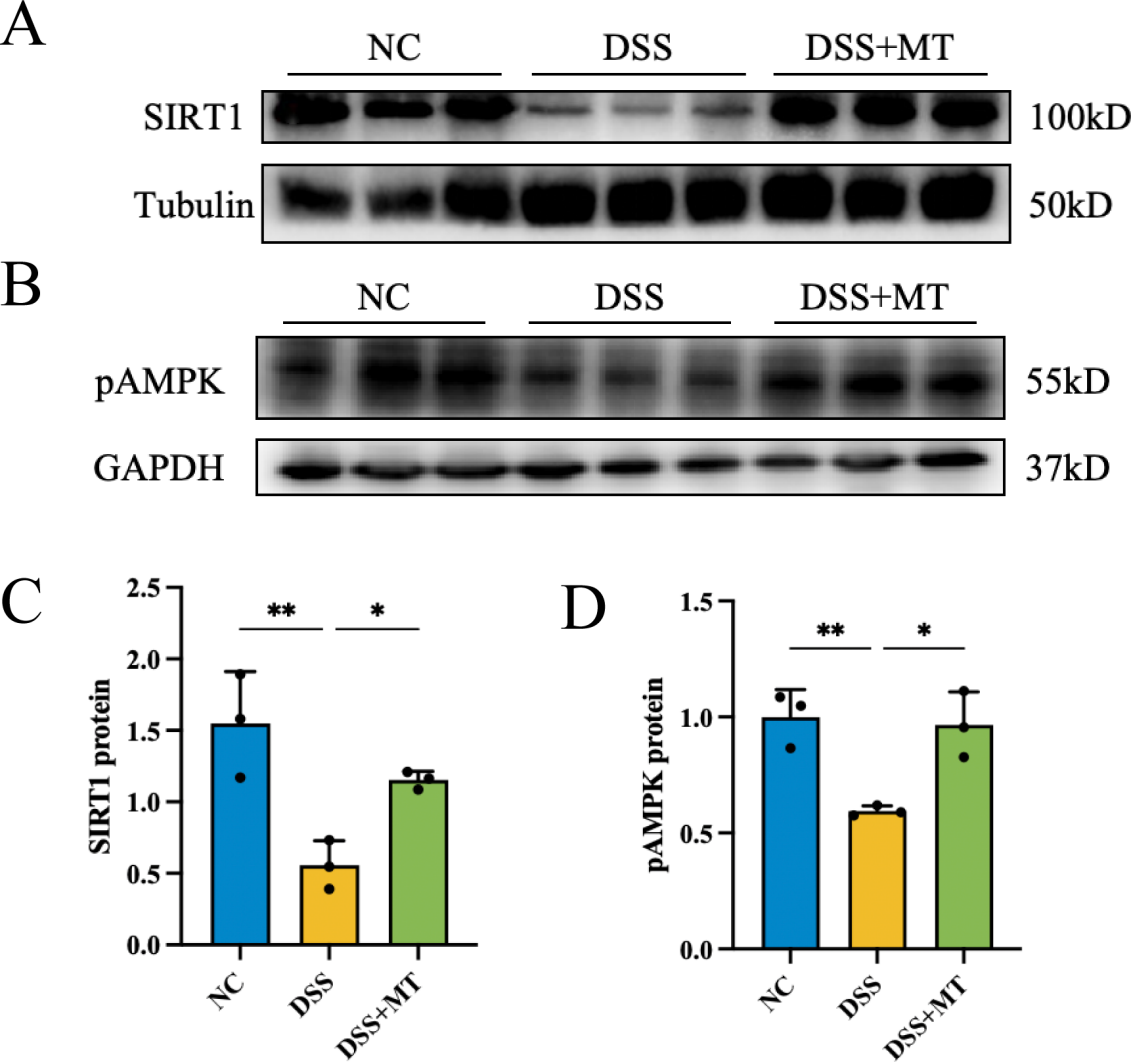
MT restored the protein levels of SIRT1 and pAMPK, n=3 (all groups). **(A, C)** The image of WB of SIRT1 protein. **(B, D)** The image of WB of pAMPK protein. (*p < 0.05, **p < 0.01).

We performed WB analysis to quantify the expression levels of SIRT1 and pAMPK in colonic tissues from the mice in Results 3.2. As shown in **Figures 5A–D**, both SIRT1 and pAMPK expression were significantly suppressed in the DSS group. Following MT intervention, the expression levels of SIRT1 and pAMPK were markedly restored. These observations suggest that the SIRT1-pAMPK axis may be involved in the protective effects of MT on the intestinal mucosal barrier, although the hierarchical relationship among these signaling components remains to be further elucidated.

### 3.5 SIRT1/pAMPK inhibitors abrogate MT-mediated colitis protection

**Figure 6:**
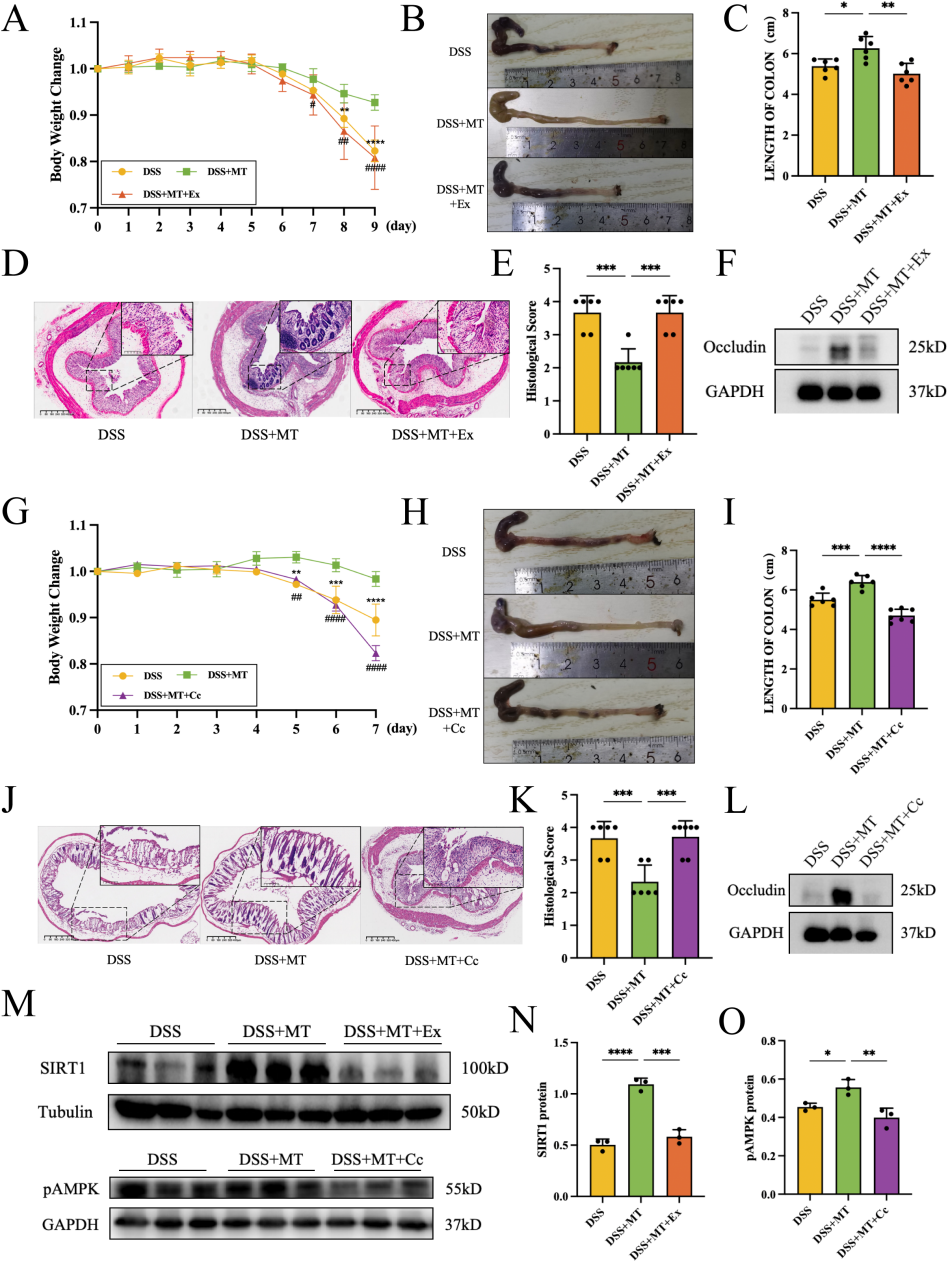
MT ameliorated colitis in mice through SIRT1/pAMPK pathway. For body weight, colon length, and H&E histopathological scoring: n=6 (DSS), n=6 (DSS+MT), n=6 (DSS+MT+Ex), n=7(DSS+MT+Cc). For qPCR and Western blotting analyses: n=3 per group. **(A, G)** The image of mice body weight changes. * indicates DSS group compared to DSS+MT group, # indicates DSS+MT+Ex/Cc group compared to DSS+MT group. **(B, C, H, I)** The length of mice colon. **(D,E, J, K)** H&E staining of colon tissues and histopathological scoring. **(F, L)** The images of WB of Occludin protein. **(M–O)** The images of WB of SIRT1 and pAMPK protein. (*p < 0.05, **p < 0.01, ***p < 0.001, ****p < 0.0001; #*p* < 0.05, ##*p* < 0.01, ####*p* < 0.0001).

To further explore the potential involvement of the SIRT1/pAMPK pathway in mediating the effects of MT, we reconstructed the DSS colitis mice model and established additional experimental groups: a SIRT1 inhibitor (Ex-527) group and an pAMPK inhibitor (Compound C) group. As shown in **Figures 6M–O**, SIRT1 expression was significantly decreased in the DSS+MT+Ex group, suggesting successful modeling in the SIRT1 inhibitor group. In the DSS+MT+Cc group pAMPK expression decreased significantly, suggesting successful modeling in the pAMPK inhibition group.

Similar to the DSS group, the body weight of mice in the DSS+MT+Ex/Cc group also showed a significant decreasing trend (**Figures 6A,G**). The length of colon was significantly shortened in the DSS+MT+Ex/Cc group (**Figures 6B,C, H,I**). The histomorphology of DSS+MT+Ex/Cc group was closer to that of DSS group (**Figures 6D,E, J,K**). The colonic histomorphological scores were analysed on HE stained images, and the scores of the DSS+MT+Ex/Cc group were similar to those of the DSS group. It was further confirmed by WB that the expression of colonic tissue tight junction protein Occludin was significantly lower in the DSS+MT+Ex/Cc group (**Figures 6F, L**). This finding suggests that inhibition of SIRT1 or pAMPK attenuates the MT-associated increase in Occludin expression, further supporting a link between this signaling axis and tight junction regulation.

**Figure 7:**
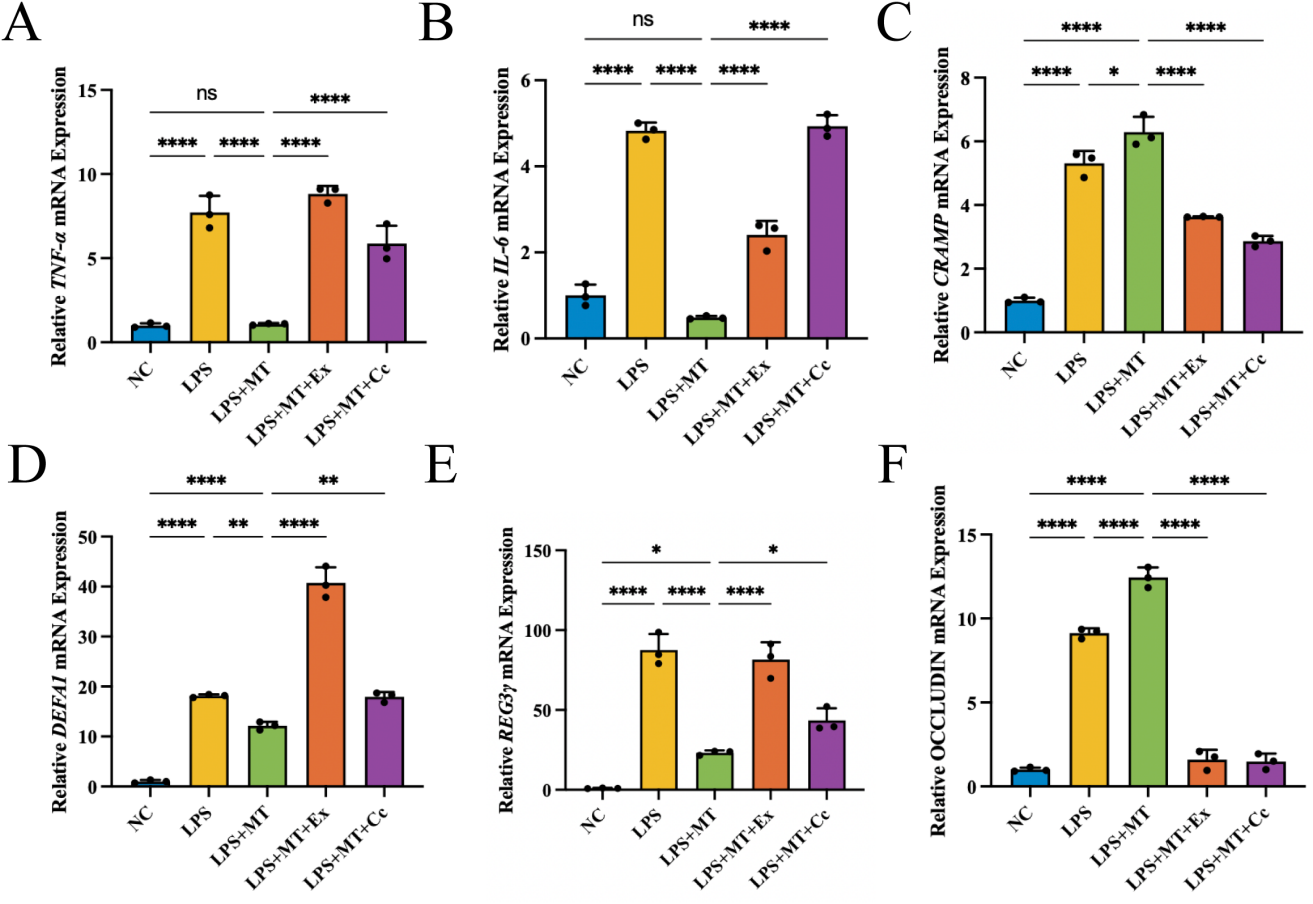
MT ameliorated LPS-induced inflammation *in vitro* through SIRT1/pAMPK pathway, n=3 (all groups). **(A, B)** The relative mRNA expression levels of proinflammatory factors in NCM460 cells. **(C–E)** The relative mRNA expression levels of antimicrobial peptides in NCM460 cells. **(F)** The relative mRNA expression levels of tight junctions in NCM460 cells. (ns *p* > 0.05, *p < 0.05, **p < 0.01, ****p < 0.0001).

Similarly, we also constructed the LPS inflammation model of NCM460 cells again with MT intervention and blocked the corresponding pathways with Ex-527 and Compound C. As shown in **Figures 7A,B**, the inhibitory effect of MT on inflammatory factors (*TNF-ɑ* and *IL-6*) disappeared after Ex-527/Compound C intervention. As shown in **Figures 7C–E**, the antimicrobial peptide *CRAMP* was further upregulated after MT treatment, whereas the expression of *DEFA1* and *REG3γ* was significantly reduced in the LPS+MT group compared with the LPS group. All these modulations were blocked by Ex-527/Compound C. *In vitro*, OCCLUDIN expression in the LPS group showed a significant elevation compared with the NC group, which we interpreted as a compensatory response. This expression level was further elevated after MT intervention, suggesting a potential association between MT treatment and enhanced tight junction expression. The MT-associated elevation of OCCLUDIN expression was suppressed by Ex-527 or Compound C (**Figure 7F**), further indicating that SIRT1 and pAMPK may be involved in this regulatory process, although mechanistic dependence remains to be directly demonstrated.

### 3.6 MT attenuates colitis via the SIRT1-LKB1-pAMPK axis

**Figure 8:**
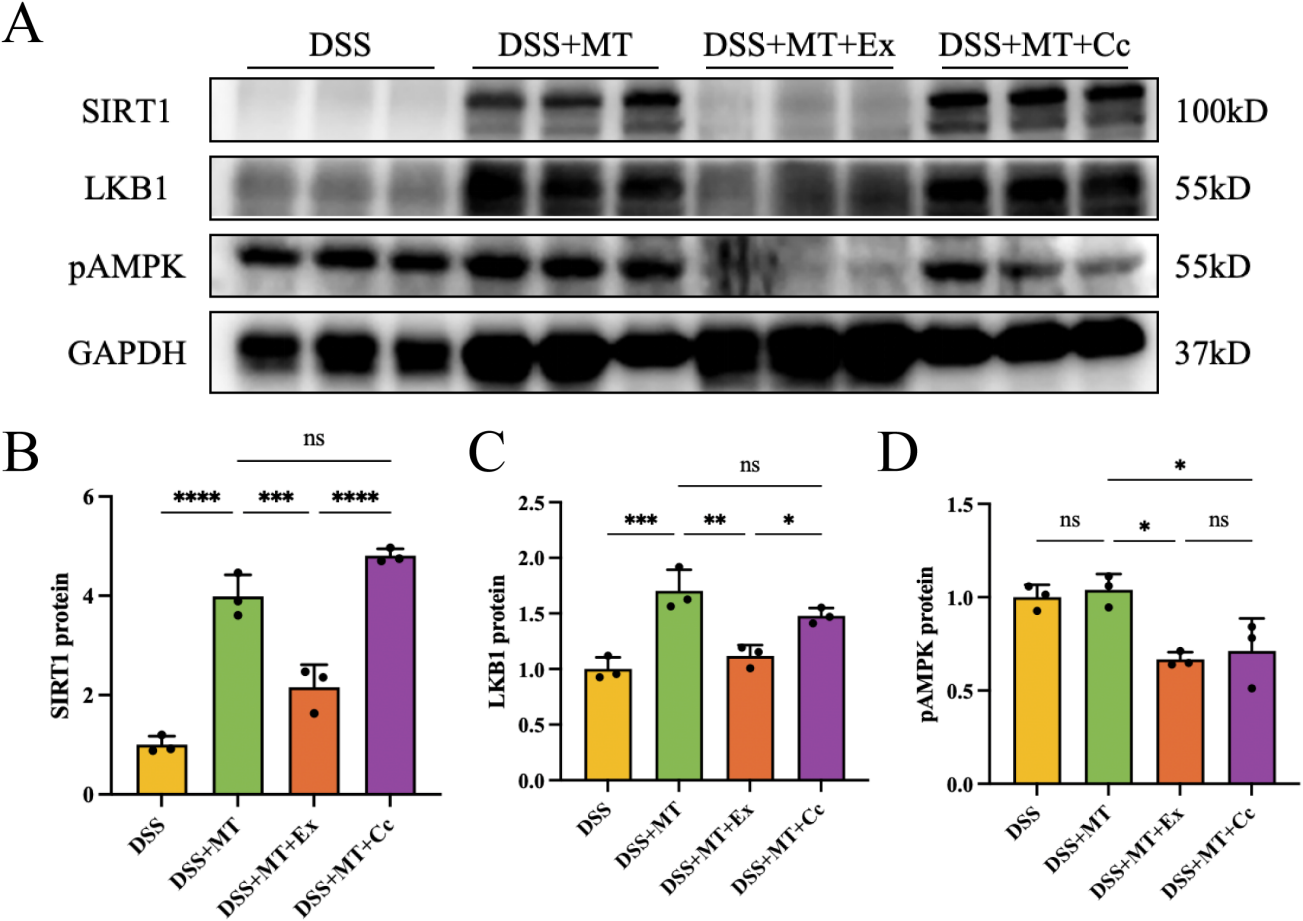
MT inhibited inflammation in colitis via SIRT1-LKB1-pAMPK pathway, n=3 (all groups). **(A)** The image of WB of SIRT1, LKB1 and pAMPK protein. **(B–D)** The image of quantitative analysis of SIRT1, LKB1 and pAMPK expression levels. (ns *p*>0.05, *p < 0.05, **p < 0.01, ***p < 0.001, ****p < 0.0001).

MT ameliorated inflammation through activation of SIRT1 and pAMPK pathways, modulating antimicrobial peptides and enhancing colonic tight junctions, which demonstrated a high level of consistency. To clarify the specific role of MT in this regulatory process, we further performed WB assays of SIRT1, LKB1 and pAMPK proteins in the animal colonic tissue. As shown in **Figures 8A–D**, after inhibition of SIRT1 (DSS+MT+Ex), the expression of LKB1 and pAMPK proteins was also inhibited. After inhibition of pAMPK (DSS+MT+Cc), the expression of SIRT1 and LKB1 proteins was not affected. Taken together, these findings suggest that MT may improve the colonic mucosal barrier through a pathway involving SIRT1, LKB1 and pAMPK, although direct evidence for the hierarchical ordering of these molecules is not provided in this study.

## 4 Discussion

Growing evidence suggests that circadian rhythm disturbance affects the risk of IBD. In this study, we demonstrated that IBD patients had decreased serum MT levels and fecal 2-oxomelatonin levels. We elucidated MT enhances the function of intestinal mucosal barrier and restores the intestinal microbiota dysbiosis via SIRT1-LKB1-pAMPK axis, eventually diminished the intestinal inflammation.

A previous study has revealed that plasma MT levels were significantly lower in UC patients than in healthy controls (19). One of the major metabolic pathways of MT is through Cytochrome P450 1A2 (CYP1A2) to eventually form 6-Sulfatoxymelatonin (6-SMT), which is the main metabolite of MT in urine (25). The UC patients exhibited a significantly lower level of urinary 6-SMT, which was correlated with the severity of UC (26, 27). To further investigate the metabolic signature of MT in the gastrointestinal tract, we analysed a larger sample size of fecal specimens by untargeted metabolomics sequencing. We observed the levels of 2-oxomelatonin were significantly lower in IBD patients compared to HCs. Compared to serum MT, fecal 2-oxomelatonin is more sensitive to changes in the gut microenvironment. 2-oxomelatonin may have the potential to be a non-invasive biomarker as an indicator for IBD activity assessment (28). In addition, the data from the CD group in this study fill the gap in the currently reported studies on the changes in the level of MT and its metabolites in CD patients. Collectively, in this exploratory analysis, we observed that IBD patients appeared to have lower levels of serum MT and fecal 2-oxomelatonin compared to HCs, consistent with existing literature reports, although the sample size was limited. However, the clinical findings should be interpreted as preliminary exploratory observations that provide a rationale for the mechanistic studies.

In this study, we constructed a model of MT ameliorating DSS-induced colitis in mice. Only a limited number of studies have addressed that MT ameliorates DSS colitis in mice despite the differences in the drug doses and intervention times used for MT and DSS (29, 30). However, it should be noted that the acute DSS-induced colitis model used in this study primarily reflects epithelial injury rather than the chronic, immune-driven pathophysiology of human IBD (31). Additionally, only male mice were included, which limits the generalizability of the findings regarding potential sex differences in IBD pathology or melatonin signaling.

Consistent with the results of our study, up-regulation of pro-inflammatory factors (TNF-α, IL-1β, IL-6 and IL-17), which can contribute to the increase of intestinal inflammation, cause damage to the intestinal mucosal barrier, and even cause systemic inflammatory responses, can be observed in the DSS model of colitis (32). One of the important pathological aspects of IBD is the migration of CD4+ T cells to the intestinal lamina propria to exacerbate the intestinal inflammatory response. This migration is enhanced by pro-inflammatory factors secreted by innate immune cells such as TNF-α, IL-1β, and IL-6 (33). IL-6 can enhance the activation of NF-κB in intestinal epithelial cells, which further upregulates the level of intercellular adhesion molecule-1 and thus promotes the recruitment of neutrophils to this area and further exacerbates inflammation (34). The study of MT ameliorating intestinal dysbiosis in stressed mice indicated that MT inhibited the activation of NF-κB and further suppressed the production of TNF-α, IL-1β and IL-6 (35, 36). In summary, MT can improve intestinal inflammation by inhibiting the expression of pro-inflammatory factors.

Antimicrobial peptides are a class of small molecule peptides with broad-spectrum antimicrobial activity, directly killing or inhibiting microorganisms such as bacteria, fungi, viruses, etc., as well as regulating immune responses and maintaining intestinal flora homeostasis (37). The results of antimicrobial peptide revealed distinct regulatory patterns following MT treatment that warrant further discussion. *Cramp* was consistently upregulated by MT in both *in vivo* and *in vitro*. CRAMP has an important role in protecting the intestinal mucosa. Lack of *Cramp* will cause severe colitis (38), whereas colonic epithelial cell damage can be effectively alleviated with CRAMP supplementation (39). The consistent upregulation of *Cramp* by MT suggests a direct protective effect of melatonin on colonic epithelial cells, which is consistent with previous findings that melatonin administration increases antimicrobial peptide production in intestinal epithelial cells (30). In contrast, *Defa1* and *Reg3γ* were downregulated by MT in both models. Defensin α (DEFA) is a class of α-defensins known in the human body as human neutrophil peptide (HNP) because it is mainly produced by neutrophils (40). The infiltration of neutrophils in the intestinal mucosa of IBD patients can lead to the upregulation of regional HNP1 and HNP3 levels in the intestinal mucosa (41). The elevation of HNP1 may lead to more significant inflammatory damage (42). In contrast, critically ill patients with low *Defa3* gene expression may be more susceptible to hospital acquired infections (43). DEFA4 acts similarly to *Defa3 (*40). Therefore, the downregulation of *Defa1* by MT may reflect reduced neutrophil recruitment and attenuation of intestinal inflammation. Regenerating Islet-Derived 3 (REG3) belongs to the C-type lectin family (40). REG3β exhibits a protective effect against intestinal translocation of gram-negative *Streptococcus* enterocolitica in mice (44). Downregulation of *Reg3γ* can be explained by the reduction in gram-negative bacteria. Our 16S sequencing data showed that MT significantly reduced the abundance of *Desulfovibrio*, a gram-negative genus. Since LPS from gram-negative bacteria stimulates *Reg3γ* expression via TLR4 signaling, the MT-induced suppression of gram-negative bacteria would lead to reduced *Reg3γ* expression (30). Consistently, *Reg3γ* was reflected to be significantly up-regulated in the DSS group and reduced after fecal transplantation (45). Collectively, these findings indicate that MT exerts multifaceted regulation of different antimicrobial peptide (AMP) classes—upregulating protective *Cramp* while downregulating inflammation-associated *Defa1* and gram-negative bacteria-induced *Reg3γ*—collectively contributing to the amelioration of colitis.

In summary, MT can modulate the expression of a variety of antimicrobial peptides to improve the colonic intestinal mucosal barrier. The common members of tight junction proteins include the Claudins family, ZO-1 and Occludin. ZO-1 is a cytoplasmic peripheral membrane protein that mediates interactions between proteins, while Occludin is a transmembrane protein with an important role in the intercellular junction complex (46). Consistent with previous findings, our study similarly observed downregulated ZO-1 and Occludin expression in intestinal epithelial injury, with subsequent recovery following therapeutic intervention (47–49). It is important to note, however, that these data demonstrate associations between MT treatment and tight junction protein expression rather than proving a direct causal relationship using functional permeability measurements or tissue-specific genetic manipulations. Future studies using intestinal epithelial cell-specific SIRT1 or pAMPK knockout models and direct functional permeability assays, such as fluorescein isothiocyanate (FITC)-dextran flux, are warranted to confirm a causal role of this signaling axis in MT-mediated barrier protection.

MT modulates the intestinal flora of DSS colitis mice. α Diversity can be assessed by Shannon, Simpson and chao1 diversity indices. In contrast to the trend of our study, the studies of Yu et al. and Xu et al. noted that the Shannon diversity index of the intestinal flora of mice in the DSS group decreased and rebounded after treatment (50, 51). As for the trend of Simpson’s index, some studies have suggested that DSS causes it to decrease (51), while others have suggested that it increases (52). We came up with similar results with most of the studies, with a decrease in Chao1 index in the DSS group compared to the NC group. The Chao1 index in the DSS+MT group in our study showed a non-significant change due to the large intra-group variation, however, most of the studies suggest a reversion of the Chao1 index in the treatment group (50). These differences of the results from the previous studies may be caused by factors such as DSS drug concentration, length of intervention, and individual differences in mice. Similar results to ours for the isolation of flora structures were obtained in the study by Xie et al (53). It can be seen that DSS led to changes in the structure of the intestinal flora of mice, while MT regulated the structure of the intestinal flora to a certain extent and brought it closer to the NC group.

In this study, the sequencing results were further classified and annotated, and the relative abundance at the phylum and genus level was statistically analysed. Of interest, the relative abundance of *Verrucomicrobia* was reduced in the DSS group, whereas the abundance of this phylum was substantially increased after MT treatment. *Akkermansia* belongs to the *Verrucomicrobia*, which was originally isolated from the feces of healthy adults and is a gram-negative anaerobic bacterium. Its subordinate species are mainly *Akkermansia*, whose abundance is reduced in the fecal flora of patients with IBD (54). It has been suggested that the reduction of Akkermansia can be one of the markers of dysbiosis in UC patients (55). *Akkermansia* regulates the concentration of short-chain fatty acids, which modulate immunity and inhibit the secretion of pro-inflammatory factors to ameliorate colitis (56). *Akkermansia* also promotes healing of intestinal epithelial damage (57). Therefore, we hypothesized that MT in the present study may have ameliorated DSS colitis in mice by upregulating *Akkermansia* abundance. In addition, *Desulfovibrio* spp. are a group of strictly anaerobic gram-negative sulfate-reducing bacteria that can reduce sulfate to hydrogen sulfide by oxidizing organic matter. Overproliferation of *Desulfovibrio* may lead to accumulation of hydrogen sulfide toxins, disrupting mitochondrial function in intestinal epithelial cells, causing excessive oxidative stress and promoting an inflammatory response (58). Rowan et al. observed a significant increase in *Desulfovibrio* in the intestinal flora of UC patients and suggested that this was associated with acute phase UC (59). Reduced abundance of *Desulfovibrio* was also found to ameliorate intestinal inflammation in mice (60). Thus, MT may also ameliorate DSS colitis in mice by inhibiting *Desulfovibrio*. Correlation analyses further revealed that *Akkermansia* abundance was negatively associated with pro-inflammatory cytokine expression and positively associated with tight junction protein expression, whereas *Desulfovibrio* showed the opposite pattern. These associations support the potential functional relevance of these microbial changes in the context of colitis. However, the causal role of the microbiota in mediating the effects of melatonin remains to be established through functional studies such as fecal microbiota transplantation in germ-free mice.

Our subsequent experiment verified that MT improves the colonic intestinal mucosal barrier by activating the SIRT1-LKB1-pAMPK axis. The activity of SIRT1 is often suppressed in inflammation-induced diseases, and several pharmacological experiments suggest that SIRT1 agonists can effectively mitigate inflammatory injury (61). Importantly, the SIRT1-pAMPK signaling axis is known to intersect directly with inflammatory pathways. pAMPK activation has been shown to suppress IκB Kinase (IKK)/IκB/NF-κB signaling, a central pathway driving pro-inflammatory cytokine production (62). Specifically, pAMPK can directly phosphorylate IKKβ, thereby inhibiting its activity and reducing NF-κB nuclear translocation. Similarly, SIRT1 can deacetylate the NF-κB p65 subunit at lysine 310, which attenuates its transcriptional activity and reduces the expression of pro-inflammatory cytokines (63). It has been reported that MT attenuates diabetes-induced renal impairment by activating the pAMPK-SIRT1 signaling pathway (64). Thus, the MT-induced activation of the SIRT1-LKB1-pAMPK axis observed in our study may contribute to the suppression of intestinal inflammation at least in part through inhibition of NF-κB-mediated inflammatory signaling. Mechanistically, SIRT1 is unable to directly activate pAMPK. Therefore, we examined LKB1, an upstream kinase of pAMPK. We observed upregulated expression of LKB1 in the colonic epithelium following MT supplementation, while SIRT1 inhibitor reduced LKB1 protein levels. Taken together, these findings suggest that MT may exert its protective effects on the intestinal mucosal barrier through activation of the SIRT1-LKB1-pAMPK pathway. However, direct molecular evidence between these components, such as gain-of-function experiments or co-immunoprecipitation assays, remain to be validated in future studies.

The upstream mechanisms by which melatonin activates SIRT1 warrant further discussion. Melatonin has been shown to increase intracellular NAD+ levels, a critical cofactor for SIRT1 activity, potentially through upregulation of the rate-limiting enzyme in the NAD+ salvage pathway (65). Additionally, melatonin can indirectly activate SIRT1 through pAMPK, as pAMPK activation increases NAD+ levels and enhances SIRT1 activity, forming a positive feedback loop (66). Some studies have also suggested that melatonin may signal through its membrane receptors (MT1/MT2) to regulate downstream molecules including SIRT1 (67). Furthermore, melatonin may indirectly preserve SIRT1 activity by reducing oxidative stress, which is known to impair SIRT1 function (68). However, whether melatonin directly modulates SIRT1 enzymatic activity or acts primarily through metabolic adaptation remains to be fully elucidated. Furthermore, melatonin’s well-documented antioxidant and mitochondrial protective actions (69) may intersect with or act in parallel to the SIRT1-pAMPK axis, given the established crosstalk between redox status, mitochondrial function, and SIRT1/pAMPK signaling. Disentangling these interconnected pathways represents an important direction for future mechanistic studies.

Several limitations should be acknowledged in this study. The clinical cohort was small and lacked detailed clinical parameters, and direct molecular evidence for the SIRT1-LKB1-pAMPK axis is not provided. The acute DSS-induced colitis model primarily reflects epithelial injury and does not fully recapitulate human IBD; additionally, only male mice were included, and sex differences were not explored. The *in vitro* experiments relied on LPS stimulation of NCM460 cells, which, despite its common use in epithelial models, does not fully capture the complex inflammatory environment in gut of IBD patients.

## 5 Conclusion

Taken together, our findings suggest that MT may ameliorate colitis by regulating gut microbiota, modulating antimicrobial peptide secretion, and reinforcing intestinal epithelial barrier integrity, potentially via activation of the SIRT1-LKB1-pAMPK pathway. These observations provide a preliminary basis for further investigation into MT as a therapeutic strategy for IBD, although clinical validation is required.

## Data Availability

The 16S rDNA amplicon sequencing data presented in this study can be found in the NCBI repository with accession number PRJNA1449969 (SRA).

## Supplementary material

Supplementary Figure S1 MT altered relative abundances of *Akkermansia* and *Desulfovibrio*, n=5 (all groups). **(A)** Relative abundance of *Akkermansia* in the NC (C), DSS (D), and DSS+MT (M) groups. **(B)** Relative abundance of *Desulfovibrio* in the three groups. (ns *p*>0.05, **p* < 0.05).

Supplementary Figure S2 Correlation analyses between key bacterial genera and inflammatory/barrier markers, n=3 (all groups). **(A, B)** Correlation plots showing significant associations between *Akkermansia* and *Tnf* and Occludin. **(C–E)** Correlation plots showing significant associations between *Desulfovibrio* and *Tnf, Il6* and Occludin. The Pearson correlation coefficient (r) and *p* are indicated for each panel.

Supplementary Table 1 Clinical characteristics of participants with serum sample collection (SD: Standard Deviation; *p* value^*^ vs HC group, with Fisher’s exact test; *p* value^†^ vs HC group, with unpaired t test or Mann-Whitney U test; n.s., not significant).

Supplementary Table 2 Clinical characteristics of participants with fecal sample collection (SD: Standard Deviation; *p* value^*^ vs HC group, with chi-square test or Fisher’s exact test; *p* value^†^ vs HC group, with Mann-Whitney U test; n.s., not significant).

Supplementary Table 3 Primers used in real-time PCR analysis. (5'-3').
