# Supplementary figures and images for: Melatonin-induced restoration of the intestinal mucosal barrier in inflammatory bowel disease via activation of the SIRT1-LKB1-pAMPK axis
Source: Front Immunol. 2026 May 5;17:1811583. doi: 10.3389/fimmu.2026.1811583 (PMC13183551; doi:10.3389/fimmu.2026.1811583)

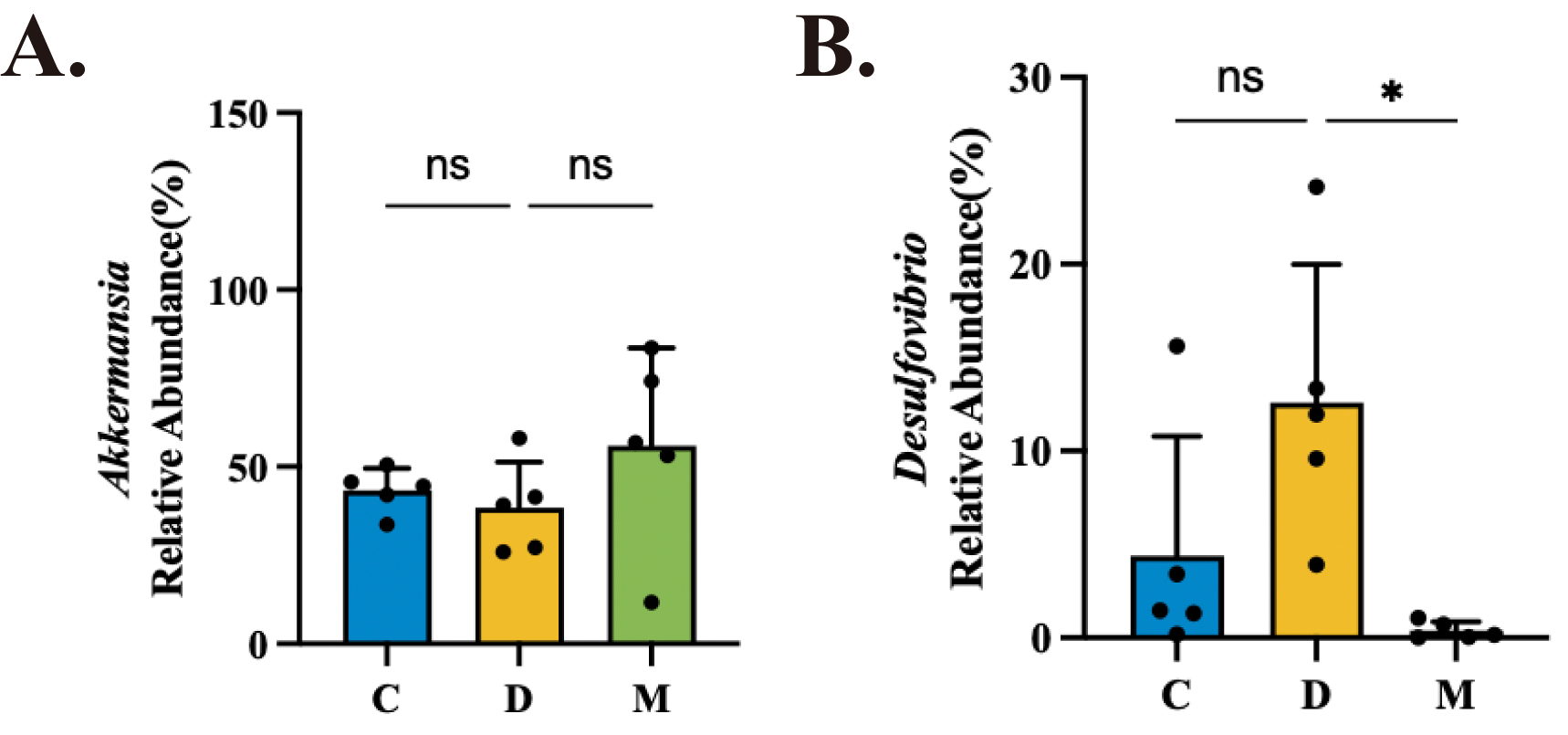

Supplement: Supplementary Figure S1 — MT altered relative abundances of Akkermansia and Desulfovibrio, n=5 (all groups). (A) Relative abundance of Akkermansia in the NC (C), DSS (D), and DSS+MT (M) groups. (B) Relative abundance of Desulfovibrio in the three groups. (ns p>0.05, *p < 0.05). [file Image1.tif]

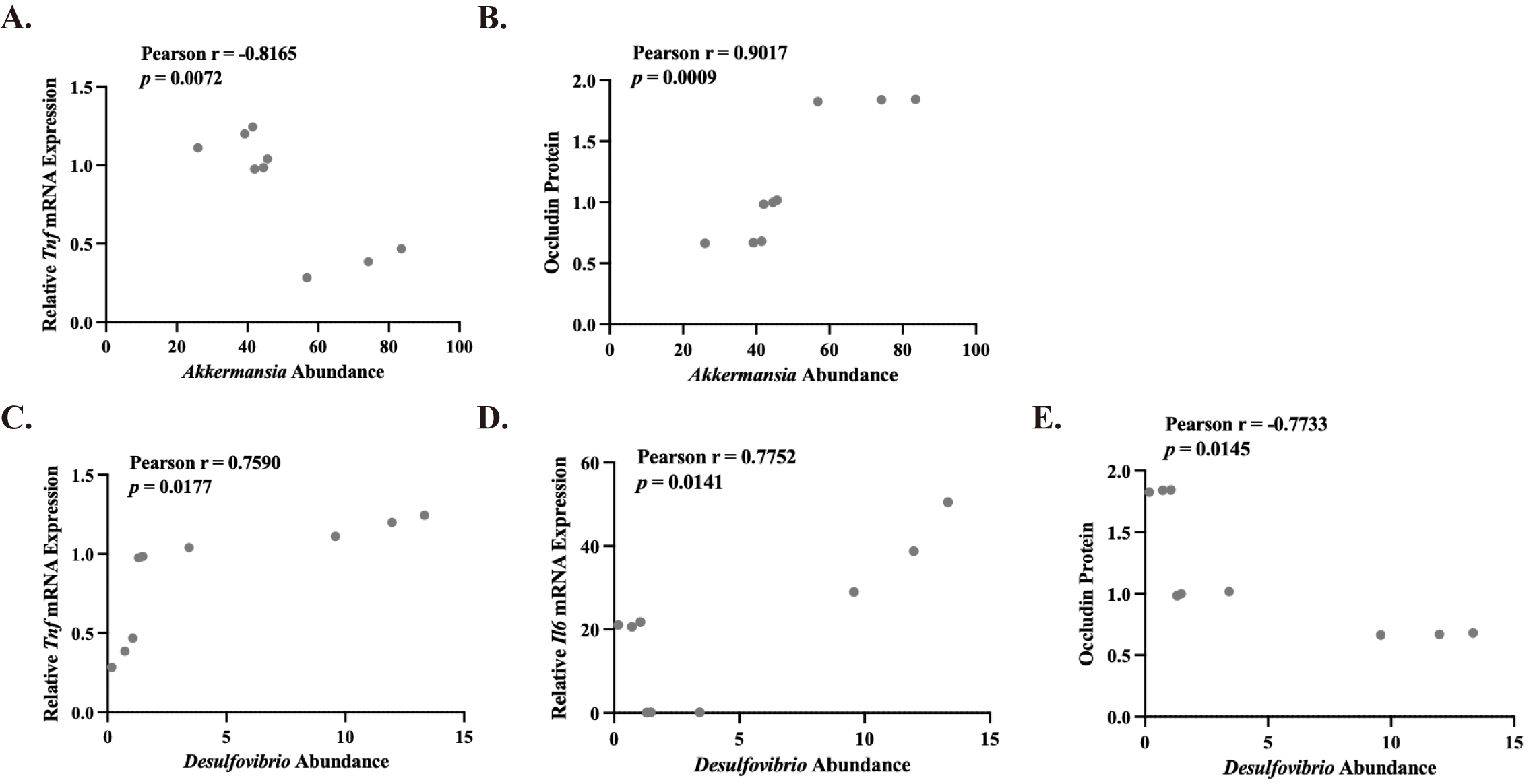

Supplement: Supplementary Figure S2 — Correlation analyses between key bacterial genera and inflammatory/barrier markers, n=3 (all groups). (A, B) Correlation plots showing significant associations between Akkermansia and Tnf and Occludin. (C–E) Correlation plots showing significant associations between Desulfovibrio and Tnf, Il6 and Occludin. The Pearson correlation coefficient (r) and p are indicated for each panel. [file Image2.tif]
