# Supplementary material for: Melatonin-induced restoration of the intestinal mucosal barrier in inflammatory bowel disease via activation of the SIRT1-LKB1-pAMPK axis
Source: Front Immunol. 2026 May 5;17:1811583. doi: 10.3389/fimmu.2026.1811583 (PMC13183551; doi:10.3389/fimmu.2026.1811583)
Supplement: Supplementary Table 1 — Clinical characteristics of participants with serum sample collection (SD: Standard Deviation; p value* vs HC group, with Fisher’s exact test; p value† vs HC group, with unpaired t test or Mann-Whitney U test; n.s., not significant). [file DataSheet1.docx]

Supplementary Material

# Supplementary Figures and Tables

**Supplementary Table1: Clinical characteristics of participants with serum sample collection (SD: Standard Deviation; *p* value^*^ vs HC group, with Fisher’s exact test; *p* value^†^ vs HC group, with unpaired t test or Mann-Whitney U test; n.s.: not significant).**

| Group | n | Sex (Male/Female) | Sex *p* value^*^ | Age(years), Mean±SD | Age *p* value^†^ |
| --- | --- | --- | --- | --- | --- |
| HC | 6 | 4/2 | - | 24.83±1.941 | - |
| UC | 6 | 4/2 | n.s. | 26.50±7.714 | n.s. |
| CD | 6 | 6/0 | n.s. | 23.33±13.17 | n.s. |
| IBD | 12 | 10/2 | n.s. | 24.92±10.42 | n.s. |

**Supplementary Table2: Clinical characteristics of participants with faecal sample collection (SD: Standard Deviation; *p* value^*^ vs HC group, with chi-square test or Fisher’s exact test; *p* value^†^ vs HC group, with Mann-Whitney U test; n.s.: not significant).**

| Group | n | Sex (Male/Female) | Sex *p* value^*^ | Age(years), Mean±SD | Age *p* value^†^ |
| --- | --- | --- | --- | --- | --- |
| HC | 26 | 15/11 | - | 42.62±16.04 | - |
| UC | 11 | 6/5 | n.s. | 51.55±9.374 | n.s. |
| CD | 21 | 15/6 | n.s. | 26.33±9.232 | *p* = 0.0001 |
| IBD | 32 | 21/11 | n.s. | 35.00±15.21 | n.s. |

**Supplementary Table3: Primers used in real-time PCR analysis. (5’-3’)**

| m-*Actb*-F | 5’-CATTGCTGACAGGATGCAGAAGG-3’ |
| --- | --- |
| m-*Actb*-R | 5’-TGCTGGAAGGTGGACAGTGAGG-3’ |
| m-*Tnf*-F | 5’-CCTGTAGCCCACGTCGTAG-3’ |
| m-*Tnf*-R | 5’-GGGAGTAGACAAGGTACAACCC-3’ |
| m-*Il1b*-F | 5’-GAAATGCCACCTTTTGACAGTG-3’ |
| m-*Il1b*-R | 5’-TGGATGCTCTCATCAGGACAG-3’ |
| m-*Il6*-F | 5’-CCAAGAGGTGAGTGCTTCCC-3’ |
| m-*Il6*-R | 5’-CTGTTGTTCAGACTCTCTCCCT-3’ |
| m-*Cramp*-F | 5’-GCTGTGGCGGTCACTATCAC-3’ |
| m-*Cramp*-R | 5’-TGTCTAGGGACTGCTGGTTGA-3’ |
| m-*Defa3*-F | 5’-CTAAAACTGAGGAGCAGCCAGG-3’ |
| m-*Defa3*-R | 5’-GCCTCTTTTTCTACAATAGCATACC-3’ |
| m-*Defa4*-F | 5’-CTAATACTGAGGAGCAGCCAGG-3’ |
| m-*Defa4*-R | 5’-GCAGTGTCCTTTTCTACAATAGCA-3’ |
| m-Reg3β-F | 5’-ACTCCCTGAAGAATATACCCTCC-3’ |
| m-Reg3β-R | 5’-CGCTATTGAGCACAGATACGAG-3’ |
| m-Reg3γ-F | 5’-ATGCTTCCCCGTATAACCATCA-3’ |
| m-Reg3γ-R | 5’-GGCCATATCTGCATCATACCAG-3’ |
| h-β-ACTIN-F | 5’-CATGTACGTTGCTATCCAGGC-3’ |
| h-β-ACTIN-R | 5’-CTCCTTAATGTCACGCACGAT-3’ |
| h-TNF-α-F | 5’-CCTCTCTCTAATCAGCCCTCTG-3’ |
| h-TNF-α-R | 5’-GAGGACCTGGGAGTAGATGAG-3’ |
| h-IL-1β-F | 5’-ATGATGGCTTATTACAGTGGCAA-3’ |
| h-IL-1β-R | 5’-GTCGGAGATTCGTAGCTGGA-3’ |
| h-IL-6-F | 5’-ACTCACCTCTTCAGAACGAATTG-3’ |
| h-IL-6-R | 5’-CCATCTTTGGAAGGTTCAGGTTG-3’ |
| h-CRAMP-F | 5’-GGCTGGTGAAGCGGTGTAT-3’ |
| h-CRAMP-R | 5’-TGGGTACAAGATTCCGCAAAAA-3’ |
| h-DEFA1-F | 5’-TCCCTTGCATGGGACGAAAG-3’ |
| h-DEFA1-R | 5’-GGTTCCATAGCGACGTTCTCC-3’ |
| h-REG3γ-F | 5’-GGTGAGGAGCATTAGTAACAGC-3’ |
| h-REG3γ-R | 5’-CCAGGGTTTAAGATGGTGGAGG-3’ |
